# Graphene Oxide-Based Silico-Phosphate Composite Films for Optical Limiting of Ultrashort Near-Infrared Laser Pulses

**DOI:** 10.3390/nano10091638

**Published:** 2020-08-20

**Authors:** Adrian Petris, Ileana Cristina Vasiliu, Petronela Gheorghe, Ana Maria Iordache, Laura Ionel, Laurentiu Rusen, Stefan Iordache, Mihai Elisa, Roxana Trusca, Dumitru Ulieru, Samaneh Etemadi, Rune Wendelbo, Juan Yang, Knut Thorshaug

**Affiliations:** 1 National Institute for Laser, Plasma and Radiation Physics, INFLPR, 409 Atomistilor Street, Magurele, 077125 Ilfov, Romania; adrian.petris@inflpr.ro (A.P.); laura.ionel@inflpr.ro (L.I.); laurentiu.rusen@inflpr.ro (L.R.); 2 National R&D Institute of Optoelectronics-INOE2000, 409 Atomistilor Street, Magurele, 077125 Ilfov, Romania; ana.iordache@inoe.ro (A.M.I.); stefan.iordache@inoe.ro (S.I.); astatin18@yahoo.com (M.E.); 3 Department of Science and Engineering of Oxide Materials and Nanomaterials, University POLITEHNICA of Bucharest, 313 Independentei Street, 060042 Bucharest, Romania; truscaroxana@yahoo.com; 4 Sitex 45 SRL, 126 A Erou Iancu Nicolae Street, 077190 Voluntari, Romania; ulierud@yahoo.com; 5 Abalonyx AS, Forskningsveien 1, 0373 Oslo, Norway; Samaneh.e@abalonyx.no (S.E.); rw@abalonyx.no (R.W.); 6 Department of Materials and Nanotechnology, SINTEF AS, Forskningsveien 1, 0343 Oslo, Norway; juan.yang@sintef.no (J.Y.); Knut.Thorshaug@sintef.no (K.T.)

**Keywords:** graphene oxide, sol-gel, silico-phosphate composite films, optical limiting functionality, ultrashort laser pulses

## Abstract

The development of graphene-based materials for optical limiting functionality is an active field of research. Optical limiting for femtosecond laser pulses in the infrared-B (IR-B) (1.4–3 μm) spectral domain has been investigated to a lesser extent than that for nanosecond, picosecond and femtosecond laser pulses at wavelengths up to 1.1 μm. Novel nonlinear optical materials, glassy graphene oxide (GO)-based silico-phosphate composites, were prepared, for the first time to our knowledge, by a convenient and low cost sol-gel method, as described in the paper, using tetraethyl orthosilicate (TEOS), H_3_PO_4_ and GO/reduced GO (rGO) as precursors. The characterisation of the GO/rGO silico-phosphate composite films was performed by spectroscopy (Fourier-transform infrared (FTIR), Ultraviolet–Visible-Near Infrared (UV-VIS-NIR) and Raman) and microscopy (atomic force microscopy (AFM) and scanning electron microscopy (SEM)) techniques. H_3_PO_4_ was found to reduce the rGO dispersed in the precursor’s solution with the formation of vertically agglomerated rGO sheets, uniformly distributed on the substrate surface. The capability of these novel graphene oxide-based materials for the optical limiting of femtosecond laser pulses at 1550 nm wavelength was demonstrated by intensity-scan experiments. The GO or rGO presence in the film, their concentrations, the composite films glassy matrix, and the film substrate influence the optical limiting performance of these novel materials and are discussed accordingly.

## 1. Introduction

The rapid progress in high-power laser sources and the numerous civilian and military applications based on them has led to an appropriate development of optical devices for protection of the human eye and sensitive optical systems.

**Figure 1:**
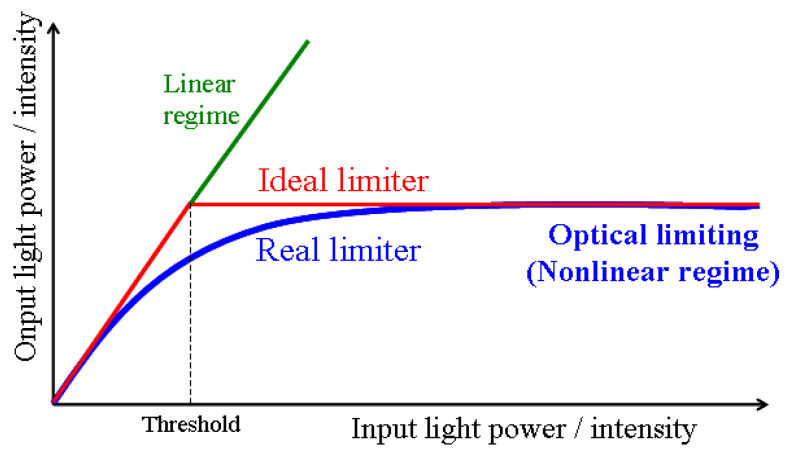
Optical limiting functionality.

Passive optical limiting (OL) functionality is based on the nonlinear optical (NLO) absorption process specific to certain NLO materials. Such an OL material shows a linear increase in the transmitted intensity/fluence of the laser beam with the incident one below a certain threshold, while above it, the transmitted intensity/fluence remains constant and independent of that of the incident laser beam. The OL functionality is schematically shown in Figure 1 for an ideal optical limiter (red curve) and for a real one (blue curve).

In Figure 1, a linear dependence of the transmitted light power/intensity on the same energetic parameters incident on the sample defines a sample characterized by a linear transmittance (green line), with no OL capability. The value of the linear transmittance, *T_L_*, is given by the slope of the corresponding linear dependency.

For a real optical limiter, the desired experimental dependencies of the transmitted light (power/intensity) on the values of the same parameters of the incident beams are not linear. The behaviour is graphically described by the blue line. This type of dependency defines the NLO transmittance, *T_NL_*, characterized by a saturation-type curve.

A wide variety of organic and inorganic materials are being studied to achieve efficient OL [1,2,3]. Graphene has been identified by many industry sectors as a key material that will drive future product development in flexible electronics, smart textiles, biosensors, drug delivery, water filtration, supercapacitors and more, as stated by the Graphene Report 2020 [4]. Lately, graphene has shown great potential as an ideal material for modern photonic, optoelectronic and electronic devices due to its ultrafast carrier relaxation dynamics and ultra-broadband NLO response as a consequence of its extended π-conjugate system and the linear dispersion relation holding for its electronic band structure [5,6,7,8,9,10,11,12,13,14].

The optical limiting in carbon-based materials, in particular, in graphene and in its derivatives, has been extensively investigated in the last years [14,15,16,17]. The optical limiting functionality of these materials, as suspension, film or bulk, has been mainly studied for visible and near-infrared nanosecond and picosecond laser pulses (for wavelengths shorter than 1100 nm) [18,19,20,21] and, to a lesser extent, for femtosecond laser pulses (mostly at 800 nm wavelength) [22,23,24,25,26]. Very few papers have investigated the nonlinear optical absorption and optical limiting of femtosecond laser pulses in the IR-B band (range, 1.4–3 μm), which includes the wavelength of 1550 nm, important for communications [27,28].

In this range of wavelengths, the solvents (water, alcohols and mixture of them) usually used for suspensions of graphene and of its derivatives have larger absorption than in the visible range, favouring the unwanted effect of bubble formation in the cells that optically limit the high-intensity laser beams.

From a practical application point of view, the transformation of the OL properties of graphene-based materials from liquid suspensions to solid-state films with a large NLO effect, low OL threshold, high damage threshold, fast response, broadband spectral response and environmental and mechanical stability is a challenging task [14,29,30].

The progress in graphene-based NLO devices, such as optical limiters, requires the preparation of optically transparent films with controlled thickness and graphene concentration [31,32]. To avoid laser damage to the system, graphene should be embedded into oxide or organic–inorganic matrices because they exhibit a higher damage threshold with respect to organic/polymers [2,33]. Sol-gel chemistry is the most suitable route for preparing homogeneous nanocomposite films from a liquid phase. A significant advantage of the method is the low material synthesis temperature. However, the design of an appropriate synthesis method for doped films via sol-gel is challenging, since the uncontrolled aggregation of the doping moieties often occurs in the precursor sol. A sol-gel synthesis route was reported for the preparation of different graphene-based silica gel glasses with optical limiting properties [29,30,34,35,36].

H_3_PO_4_ as a phosphor precursor for silica-phosphate films was reported to form Si–O–P bonds during the sol-gel process [37]. The presence of P_2_O_5_ in the reduced graphene oxide (rGO)-doped films prepared by sol-gel was reported to yield a more compact graphene-based composite layer [38].

In this paper, we describe the preparation, for the first time to the best of our knowledge, of novel graphene oxide-based silico-phosphate composite glassy materials by the sol-gel method, together with the morphology and structure characterization of the obtained films. We experimentally demonstrate, by intensity-scan experiments, the OL functionality of these NLO materials for ultrashort (~150 fs) laser pulses at the important telecommunication wavelength of 1550 nm, for which there are very few OL reported results. The influence of GO or rGO presence and of their concentrations in the silico-phosphate composite films, of the silico-phosphate matrix as well as of the film substrate, on the linear transmittance and optical limiting performance of these novel materials is discussed. We compare the OL in our samples with several OL results obtained in literature with ns, ps, and fs laser pulses at visible and near infrared wavelengths.

## 2. Experimental

### 2.1. Preparation of Silico-Phosphate Films

The sol-gel chemicals for graphene oxide-based silico-phosphate film preparation were as follows: tetraethyl orthosilicate (TEOS, 99% purity, Sigma-Aldrich, Redox Lab Supplies Com S.R.L. Bucharest, Romania) as a precursor for SiO_2_, phosphoric acid (H_3_PO_4_, 85 wt. % in H_2_O, Sigma-Aldrich, Redox Lab Supplies Com S.R.L. Bucharest, Romania) as a precursor for P_2_O_5_, and rGO/GO (as powders, supplied by Abalonyx AS, Oslo, Norway). The compositions of the starting solutions, presented in Table 1, were calculated, aiming to have different concentrations of rGO/GO in the SiO_2_-P_2_O_5_ films.

The appropriate amount of rGO/GO was dispersed in ethanol and immersed in an ultrasonic bath for 20 min. Then, TEOS was added to the suspension and magnetically stirred for 2 h before adding H_3_PO_4_. The reaction mixtures were magnetically stirred for another 24 h and afterwards spin coated onto glass and Indium Tin Oxide (ITO)-coated glass at 2000 rpm for 30 s. The thin films were thermally treated in an oven, first for drying at 200 °C with a heating rate of 5 °C/h and kept for at 200 °C and afterwards sintered at 350 °C with a heating rate of 500 °C/h and kept for 30 min at 350 °C.

**Figure 2:**
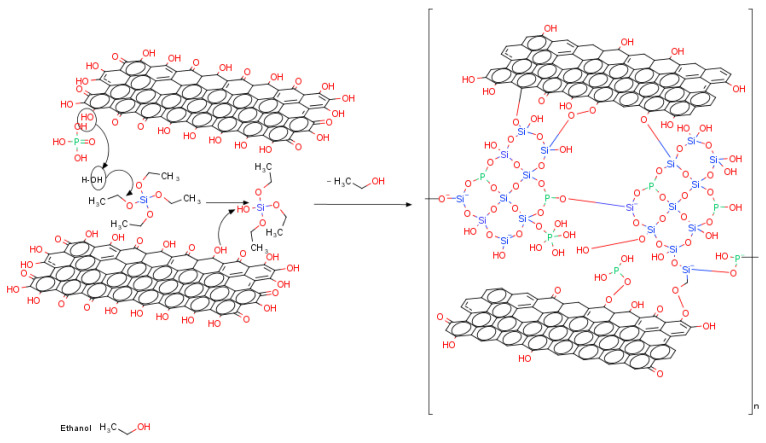
Schematic representation of the process reactions.

A proposed schematic representation of the reactions for the graphene oxide embedment into the SiO_2_-P_2_O_5_ glassy film is presented in Figure 2.

The phosphoric acid is involved in more reactions schematically presented as follows:

Hydrolyzation of TEOS: O=P(OH)_3-n_ + RO–Si– ⇌ =P–(OH)_2-n_(OSi) + R–OH; –Si–O–P– + H_2_O ⇌ –Si–OH + HO–P–

Condensation of reaction’ intermediates: –P–OH + –P–OH ⇌ –P–O–P– + H_2_O; –P–OH + –Si–OH ⇌ –P–O–Si– + H_2_O

Reaction with rGO/GO: –C–COOH + H_3_PO_4_ ⇌ –C–C=O + H_2_O + H_2_PO_4_^–^

### 2.2. Material Characterization

The chemical structure of the samples was investigated using FTIR spectroscopy (with a Spectrum 100 spectrophotometer provided with Universal Attenuated Total Reflectance (UATR) accessory (Perkin Elmer, Llantrisant, UK), in the range 550–4000 cm^−1^, with a resolution of 4 cm^−1^ and 10 scans, with Atmospheric Vapour Compensation (AVC), and Raman spectroscopy (Nicolet Almega XR, UK-Thermo Fisher Scientific, Oslo, Norway) with an excitation source of λ = 488 nm, a spot of 3 µm diameter and a power of 5 mW at the sample surface. For morphological investigations, atomic force microscopy (AFM) (XE-100 type from Park Systems, Europe GmbH, Mannheim, Germany, non-contact mode) and scanning electron microscopy (SEM) with energy-dispersive X-ray (EDX) analysis using a FEI Inspect F50 system (FEI Europe B.V. Eindhoven, Netherlands) were used. The spectral dependence of the transmittance was investigated using a UV/VIS/NIR spectrophotometer (Perkin Elmer, Lambda 1050, Llantrisant, UK).

The OL capability of the graphene oxide-based silico-phosphate films was investigated by intensity scan (I-scan) experiments [39,40,41,42,43] using ultrashort laser pulses of an Er-doped fibre laser (FemtoFiber Scientific FFS, TOPTICA Photonics AG, Munich, Germany, 1550 nm wavelength, ~150 fs pulse duration). The experimental setup is presented in the Results and Discussion section. In the OL experiments performed on different samples, the transmittance curves (transmitted pulse peak intensity vs. incident one) were determined for a range of average powers incident on the sample increasing up to the maximum value provided by the utilized laser source.

## 3. Results and Discussion

### 3.1. FTIR Spectroscopy

**Figure 3:**
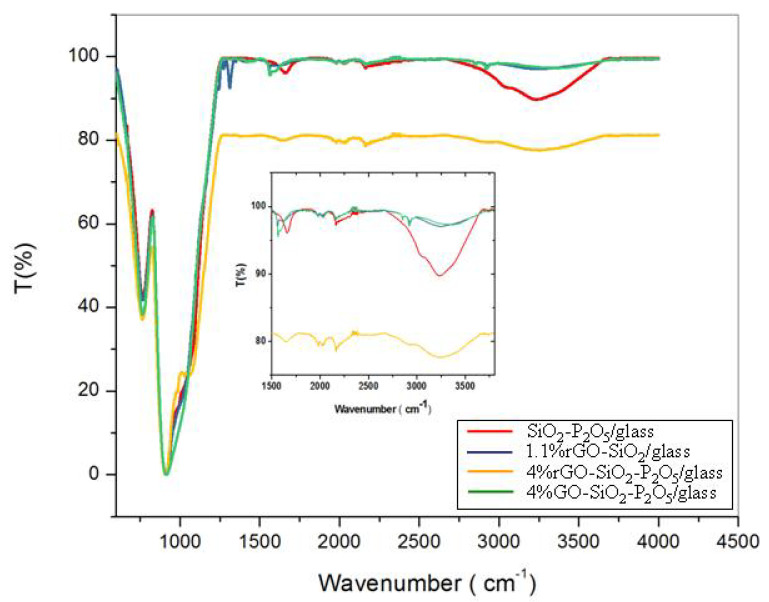
The FTIR spectra of the deposited reduced graphene oxide (rGO)-/GO-containing thin films.

The FTIR spectra are presented in Figure 3 and summarized in Table 2.

The broad peak in the region 2670–3770 cm^−1^ centred around ~3400 cm^−1^ was observed in all the samples, most pronounced for the sample (SiO_2_-P_2_O_5_) without rGO/GO, less pronounced for 1.1%rGO-SiO_2_ and even less so for samples containing P_2_O_5_. This absorption band was assigned to O–H stretching vibrations of hydroxylic, phenolic, carboxylic, P–OH groups and absorbed water molecules. For films containing P_2_O_5_, an explanation could be that H_3_PO_4_ removes the oxygen-containing functional groups (–OH, C–O and C–OH groups) from the rGO/GO structure, where the main pathway is the protonation of the OH groups followed by H_2_O elimination. This explanation is in overall agreement with the report from Er and Celikkan [44].

The two small broad peaks near ~2920 and ~2846 cm^−1^ observed in the samples with GO (4%GO-SiO_2_-P_2_O_5_) and in the ones without P–O (1.1%rGO-SiO_2_) were attributed to the stretching vibrations of C–H in –CH–OH and –CH–COOH belonging to graphene oxide and overlapping with the hydrogen-bonded OH groups of dimeric COOH groups and intra-molecular-bonded O–H stretching of alcohols, respectively [45]. Additionally, small peaks at ~1560 and ~1595 cm^−1^ visible in the FTIR spectrum of the same samples (4%GO-SiO_2_-P_2_O_5_ and 1.1%rGO-SiO_2_) were attributed to the deformation modes of absorbed water molecules’ δ(H–O–H) and O-H groups linked to the –C=O stretching vibration of carboxylic and/or carbonyl moiety functional groups and of skeletal vibrations from un-oxidized graphitic domains from rGO or GO [45,46,47].

The carboxyl stretching vibrations (C=O) at 1736 cm^−^^1^ belonging to the rGO/GO were not noticed in any of the samples. However, in the 4%rGO-SiO_2_-P_2_O_5_ sample, the large band at ~1640 cm^−1^ could be due to the shift of the C=O band towards higher wavelengths overlapping with C=C stretching vibrations.

In the 4%rGO-SiO_2_-P_2_O_5_ sample, the vibrational bands at 2920–2846 cm^−1^ were not clearly solved and a broad band centred at ~2900 cm^−1^ was noticed and attributed to the hydrogen inter-layer bonds with water molecules [48].

The large band at ~1650 cm^−1^ in the SiO_2_-P_2_O_5_ sample was attributed to OH vibrations of water molecules attached to P–O bonds.

For all the samples, the characteristic vibration bands for SiO_2_-P_2_O_5_ amorphous films were observed in the FTIR spectra: Si–O–Si symmetric stretching (~760 cm^−1^), Si–OH stretching (~910 cm^−1^) with shoulders corresponding to (TO) Si–O–P asymmetric stretching (~1060 cm^−1^), (TO) Si–O–Si symmetric stretching (~1100 cm^−1^) and (LO) Si–O–Si symmetric stretching overlapping with O–P–O symmetric stretching (~1200 cm^−1^) [49].

### 3.2. Atomic Force Microscopy

**Figure 4:**
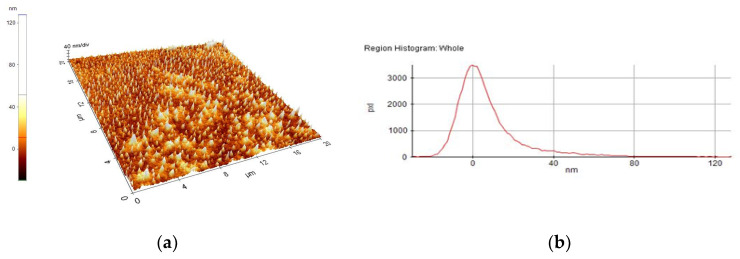
AFM image of 1%rGO-SiO_2_-P_2_O_5_/glass film: (**a**) morphology, (**b**) region histogram.

**Figure 5:**
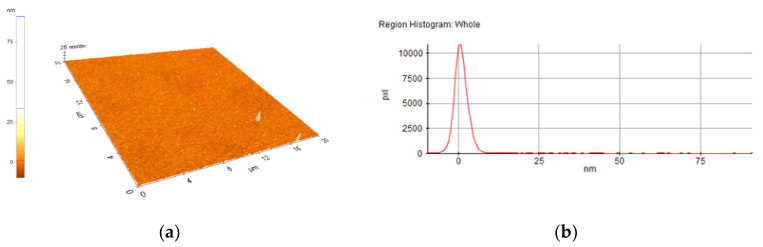
AFM image of 1%rGO-SiO_2_-P_2_O_5_/ITO film: (**a**) morphology, (**b**) region histogram

**Figure 6:**
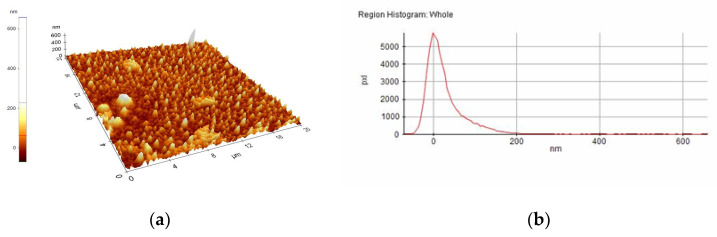
AFM image of 1.1%rGO-SiO_2_/glass film: (**a**) morphology, (**b**) region histogram.

**Figure 7:**
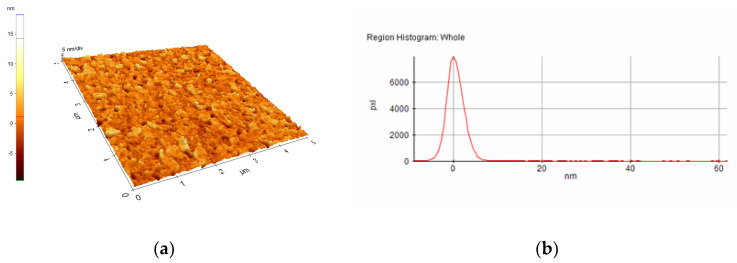
AFM image of 1.1%rGO-SiO_2_/ITO film: (**a**) morphology, (**b**) region histogram.

**Figure 8:**
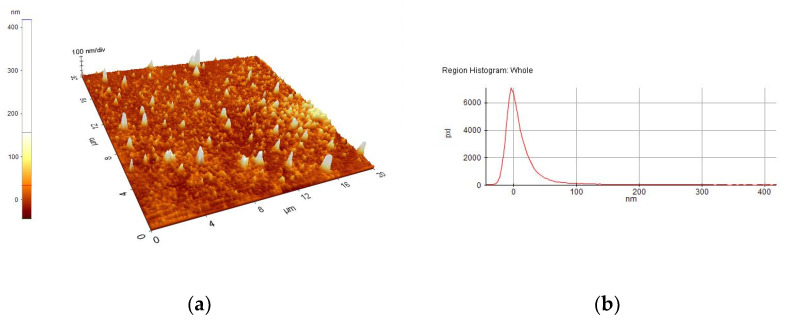
AFM image of 4%GO-SiO_2_-P_2_O_5_/glass film: (**a**) morphology, (**b**) region histogram.

**Figure 9:**
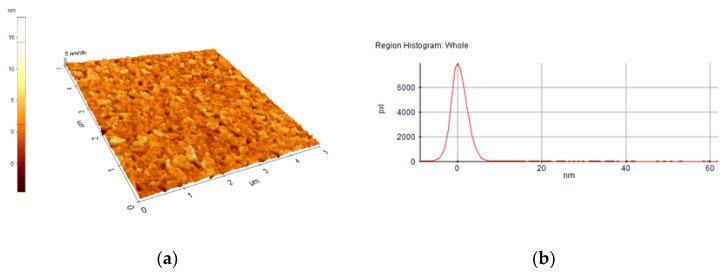
AFM image of 4%GO-SiO_2_-P_2_O_5_/ITO film: (**a**) morphology, (**b**) region histogram.

A selection of AFM images is presented in Figure 4, Figure 5, Figure 6, Figure 7, Figure 8 and Figure 9, and the values of the root-mean-squared roughness (Rq) of the films deposited on glass and ITO-coated glass are summarized in Table 3.

The prepared films were all homogenous, with the standard deviations of the height value (Rq(nm)) being in the intervals of 15–45 nm on the glass substrate and 2.2–2.4 nm on the ITO-coated glass. However, the Rpv values (Rpv is the peak-to-valley of the selected region, that is, the difference between the minimum and maximum values in the selected region) for the samples deposited on glass varied in the interval 157.5–729 nm, meaning that the pores present in the selected regions were deeper and larger for the samples without phosphor content. A more homogenous distribution of pores for the 1%rGO-SiO_2_-P_2_O_5_ samples could be seen from the distribution histograms.

For the films deposited on ITO-coated glass, the roughness was similar for samples with rGO regardless of the presence of phosphor, and the values for Rpv were similar for 1.1%rGO-SiO_2_ and 4%GO-SiO_2_-P_2_O_5_ and up to 100.3 nm for 1%rGO-SiO_2_-P_2_O_5_. As expected, the films deposited on ITO-coated glass were more compact than the ones on the glass substrate, due to the more homogeneously distributed surface-active sites of the ITO. The glass substrate is a borosilicate glass, which has a surface with low network connectivity, of the glass network formers Si and B, with the coexistence of different types of boron coordination states and with defects (i.e., a nonbridging oxygen, two-membered ring, and three-coordinated silicon) [50,51].

### 3.3. Scanning Electron Microscopy

**Figure 10:**
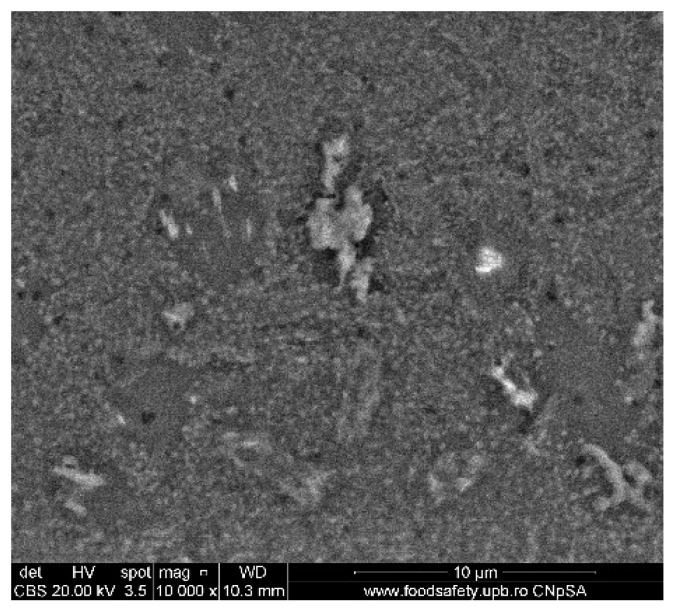
SEM image of 1.1%rGO-SiO_2_/glass.

**Figure 11:**
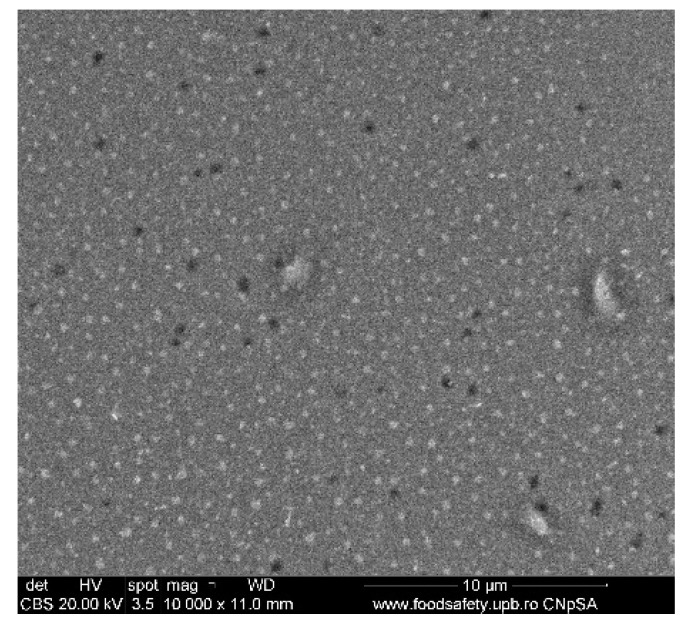
SEM image of 1%rGO-SiO_2_-P_2_O_5_/glass.

**Figure 12:**
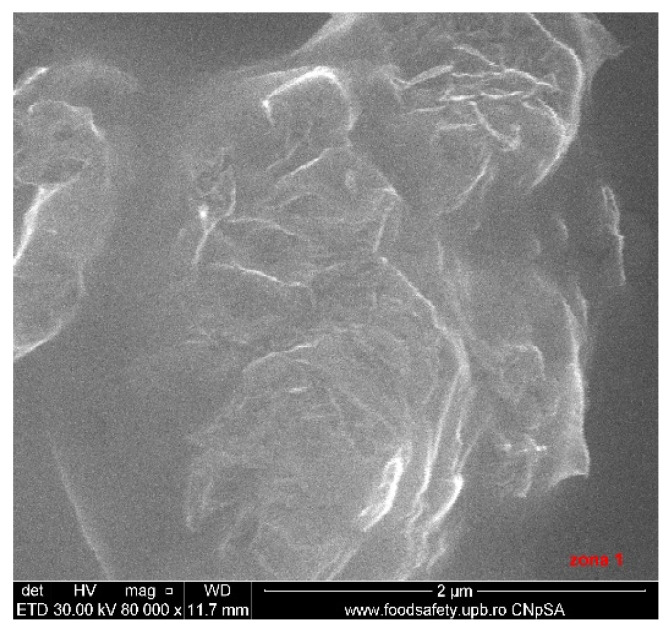
SEM image of 4%rGO-SiO_2_-P_2_O_5_/ITO.

A selection of SEM images is presented in Figure 10, Figure 11 and Figure 12. The 1.1%rGO-SiO_2_/glass film was less homogeneous than 1%rGO-SiO_2_-P_2_O_5_/glass one, as presented in Figure 10, Figure 11 and Figure 12, giving evidence of phosphorus pentoxide contribution in the distribution of rGO in the silico-phosphate matrix. This is in agreement with the AFM studies.

**Figure 13:**
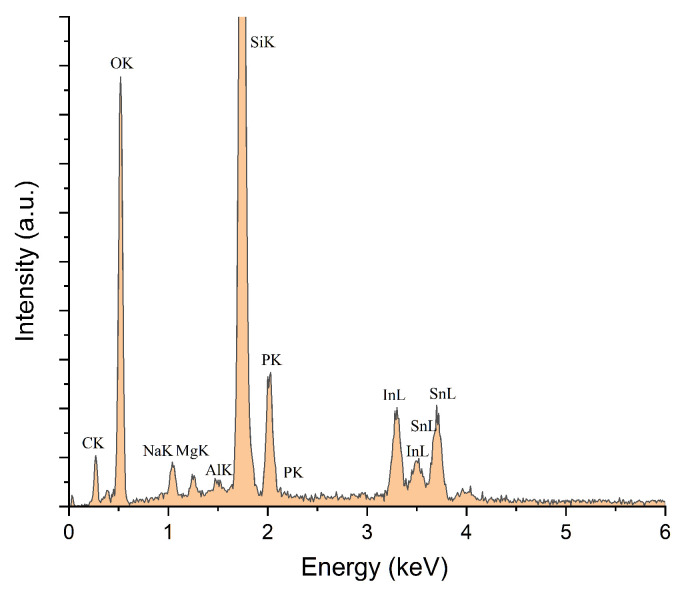
EDX spectra of 4%rGO-SiO_2-_P_2_O_5_/ITO film corresponding to the SEM image from Figure 12.

The existence of P was noticed in all samples containing P_2_O_5_, while C was noticed in the composite films more concentrated in rGO/GO, a lower GO/rGO content being under the detection limit of the equipment. This is demonstrated in Figure 13. The detailed SEM image of the 4%rGO-SiO_2_-P_2_O_5_/ITO sample and the respective EDX spectra are presented in Figure 12 and Figure 13.

### 3.4. UV-VIS-NIR Spectroscopy

**Figure 14:**
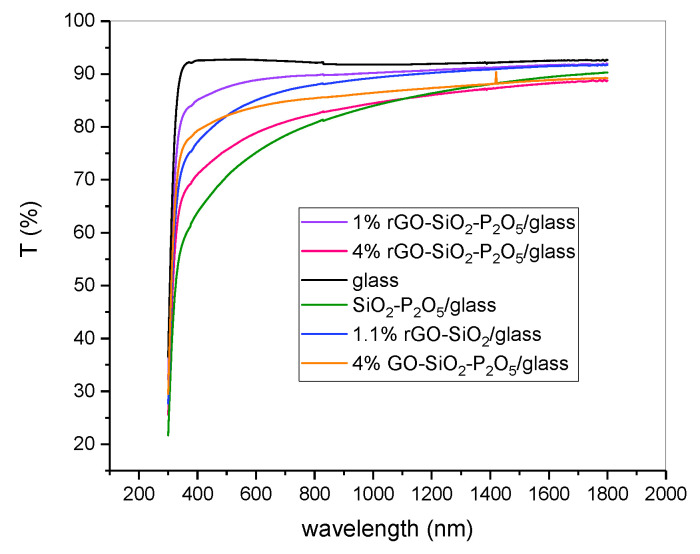
UV-VIS-NIR spectra of the sol-gel films deposited on glass substrate.

**Figure 15:**
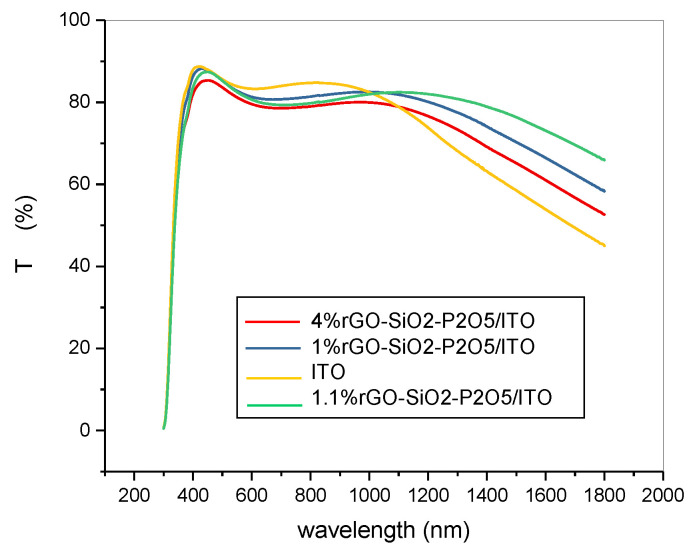
UV-VIS-NIR spectra of the sol-gel films deposited on ITO-coated glass substrate.

The UV-VIS-NIR transmission spectra of the composite sol-gel films prepared on glass and on ITO-coated glass, collected with air as reference, are presented in Figure 14 and in Figure 15, respectively.

The substrate had a strong influence on the UV-VIS-NIR spectra of the prepared samples, as revealed by comparison of the transmission spectra for the films deposited on the glass substrate (Figure 14) with the ones for the films deposited on the ITO-coated glass substrate (Figure 15).

As a general remark, in the visible domain, the transmission of the rGO/GO doped samples on glass was higher than 70%, while on the ITO-coated glass, it was higher than 80%. These films are thus suitable for the protection of sensitive equipment against a NIR laser beam, being transparent enough to see through them.

For films deposited on the glass substrate (Figure 14), the samples containing rGO/GO exhibited a transmission above 85% for λ longer than 1100 nm and followed the general trend of the SiO_2_P_2_O_5_-glass sample. For wavelengths longer than this value, the transmission decreased with increasing rGO content. For the same concentration of 4%, the film with the rGO content exhibited a higher absorbance than that of that with GO, as expected. The transmittance of the two samples with quite similar rGO content (1 and 1.1%) was higher for the sample that contained phosphorus pentoxide (P_2_O_5_) than for the sample without P_2_O_5_, for all wavelengths. The difference between the two spectra tends to be negligible for wavelengths longer than 1200 nm.

The transmittance of all the samples deposited on the ITO-coated glass substrate (Figure 15) followed the trend line of this particular type of substrate. For longer wavelengths (1100–1800 nm), the increase in rGO content induced a decrease in transmittance, as can be seen from the two spectra of the samples containing P_2_O_5_, with 1%rGO and with 4%rGO, respectively. In the same spectral range (1100–1800 nm), the transmittance of the two samples with quite similar content of rGO (1% and 1.1%) was higher for the sample without P_2_O_5,_ (1.1%rGO) than for the sample with P_2_O_5_ (1%rGO), and the difference was larger for longer wavelengths. For both films on ITO-coated glass (1.1%rGO, without P_2_O_5_) and (1%rGO, with P_2_O_5_), the transmittance did not decrease under 60% in the considered NIR spectral range.

### 3.5. Raman Spectroscopy

**Figure 16:**
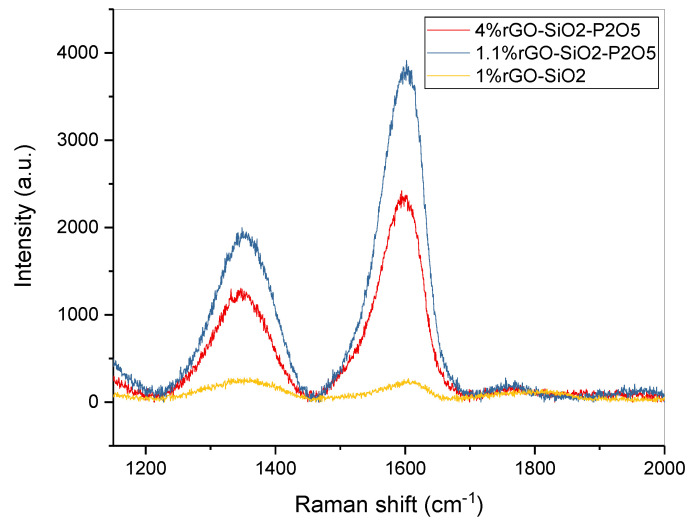
Raman spectra of a selection of the sol-gel films deposited on ITO-coated glass substrate.

The Raman spectra of the synthesized films presented in Figure 16 show the characteristic peaks for graphene derivatives, namely, the G band at approximately 1600 cm^−1^ originating from the in-plane vibration of sp^2^ carbon atoms and the D band associated with edge planes, defects and disordered structures of carbons found in graphene sheets at 1350 cm^−1^. The broad D peak suggests a highly disordered regime including the structural imperfections created by the attachment of hydroxyl and epoxide groups on the carbon basal plane [52,53,54,55].

Higher D bands were observed in samples 4%rGO-SiO_2_-P_2_O_5_ and 1.1%rGO-SiO_2_-P_2_O_5_ as compared to that in sample 1%rGO-SiO_2_, indicating a decreased disorder associated with a decreased concentration of oxygen-containing functional groups under the action of H_3_PO_4_, which contributes to a further reduction of rGO embedded in the silico-phosphate matrix. The observation is in accordance with the FTIR and AFM results.

### 3.6. Optical Limiting Capability

**Figure 17:**
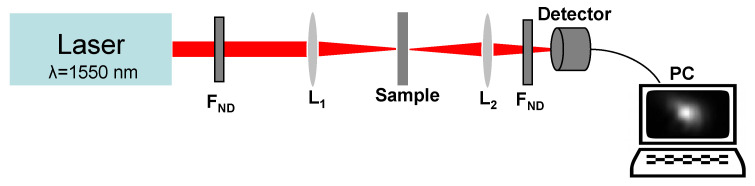
Schematic of the experimental setup for optical limiting (OL) studies.

The experimental setup used to investigate by intensity scans (I-scan) the OL capability of the synthesized graphene oxide-based silico-phosphate composite films is shown in Figure 17, and it is described below.

The laser source is an Er-doped fibre laser (Toptica), which generates ultrashort pulses (~150 fs pulse duration) with a repetition rate of 76 MHz, at the wavelength λ = 1550 nm. The maximum average power is ~230 mW, and the corresponding peak power and the pulse energy of the generated laser pulses are ~19 kW and ~3 nJ, respectively. The lens L_1_ (focal length = 2.54 cm) focuses down the laser beam on a spot of 13 μm diameter on the investigated sample, which is placed in its focal plane. The intensity of the laser beam incident on the sample is varied by changing its power only with neutral density filters (F_ND_) (Thorlabs, Munich, Germany), with the transmission specially calibrated by us at the wavelength of the laser (*λ* = 1550 nm). Using the lens L_2_, the spot size of the transmitted laser beam is adjusted relative to the aperture of the detector used to measure the beam average power. The lenses L_1_ and L_2_ and the sample are mounted on micrometric translation stages for the fine tuning of their positions. The average powers of the incident and of the transmitted beams were measured using a FieldMax II-TOP power meter (Coherent, Portland, OR, USA) with a type OP-2IR detector (Coherent, Portland, OR, USA). All the measured powers, the incident and the transmitted ones, were corrected for Fresnel reflections. Neutral density filters were also used in front of the detector in order to keep the power of the measured light signal below the maximum power measurable with the detector. The incident average laser powers in the available range are below the values for which the laser-induced damage could appear, as seen in investigations with an optical microscope.

The maximum value of the incident peak intensity in our I-scan experiments was limited by the maximum available average power of the laser source, lower than ~9 GW/cm^2^. At these incident peak intensities, no optical damage was observed in the investigated samples.

In order to investigate the OL capability of the GO/rGO composite glassy films, the values of the transmitted peak intensities, $I_{trans}(peak)$, were measured as a function of the incident peak intensities, $I_{inc}(peak)$, for the entire range of $I_{inc}(peak)$ of the available laser source. A deviation from the linear transmittance towards lower values of transmittance is indicative of the optical limiting behaviour [27,28,56].

The values of the experimentally determined transmitted peak intensities, $I_{trans}(peak)$, for incident peak intensities, $I_{inc}(peak)$, lower than ~0.5 GW/cm^2^, were fitted with a linear dependence, and from its slope, the linear transmittance, *T_L_*, of each sample was determined.

The entire set of experimentally measured transmitted peak intensities, for each investigated sample, was fitted with a saturation-type function of the form:

(1) $$I_{trans}(peak)=\frac{T_{L}\cdot I_{inc}(peak)}{1+\left[I_{inc}(peak)/I_{sat}\right]}$$

where *I_sat_* is the saturation peak intensity. This equation gives information about the overall optical limiting capability of a sample, without considering the nonlinear optical processes involved in this functionality and their contribution to the overall optical response.

As a general remark regarding the rGO/GO composite films deposited on the two considered substrates (glass and ITO-coated glass, respectively), we mention that the samples that consist of composite films deposited by spin coating on glass substrates have a higher linear transmittance in NIR than those deposited in the same conditions on ITO-coated glass substrates.

**Figure 18:**
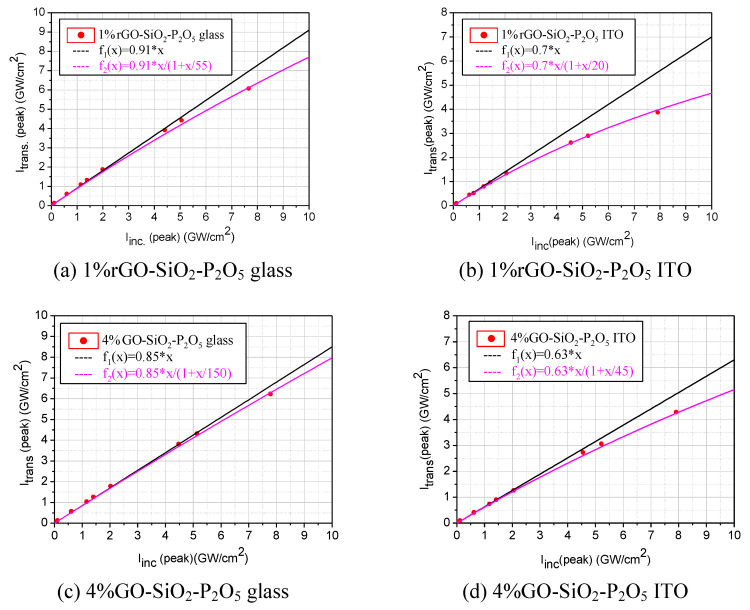
The transmitted peak intensities vs. incident ones for two sets of corresponding composite samples: with rGO deposited on glass (**a**) and on ITO-coated glass (**b**); with GO deposited on glass (**c**) and on ITO-coated glass (**d**).

This is possible to see from the experimental transmittance results, considering the incident and the transmitted peak intensities of the laser pulses, shown in Figure 18a,b (samples with rGO) and in Figure 18c,d (samples with GO), by comparing the linear transmittances and *T_L_* (slopes of linear dependencies) of the corresponding sample sets. This is consistent with the transmittances at 1550 nm from the transmission UV-VIS-NIR spectra shown in Figure 14 and Figure 15, respectively. The small differences could be due to the different incident powers (very low in the case of the spectrometer’s light source) and to the very different sizes of the illuminated areas in the two cases.

For both considered substrates, the linear transmittance is appropriate for OL functionality. OL in samples of different materials with comparable [57,58] or even much lower [56] linear transmittance has been reported.

The maximum incident peak intensities are slightly varying in the graphics shown in Figure 18a–d due to the fact that the individual optical limiting experiments were performed at different moments in time with a slightly readjusted internal optical alignment of the fs laser system, which influenced its maximum average power.

On the other hand, the OL experiments revealed the fact that the silico-phosphate composite films, based on rGO or on GO, deposited on ITO-coated glass substrates limit better (a larger deviation of the saturation-type fit of the experimental data from the straight line corresponding to linear transmittance) than those deposited on glass substrates, as observed from Figure 18a,b (samples with rGO) and in Figure 18c,d (samples with GO) of the corresponding sample sets.

The better limiting in films deposited on the ITO-coated glass substrate is probably due to the already-mentioned fact that these films are more compact than the ones deposited on the glass substrate since the active sites on the ITO surface are more uniformly distributed. For the glass substrate, the phosphorus pentoxide content in the films contributes to the decrease in the pore size of the deposited composite films.

In order to compare the influence of the rGO and GO, respectively, and of their concentrations, and to assess the influence of the silico-phosphate matrix on the OL performance of the composite films, in the following, we will show and analyse the results obtained on the composite films deposited on ITO-coated glass substrates only.

**Figure 19:**
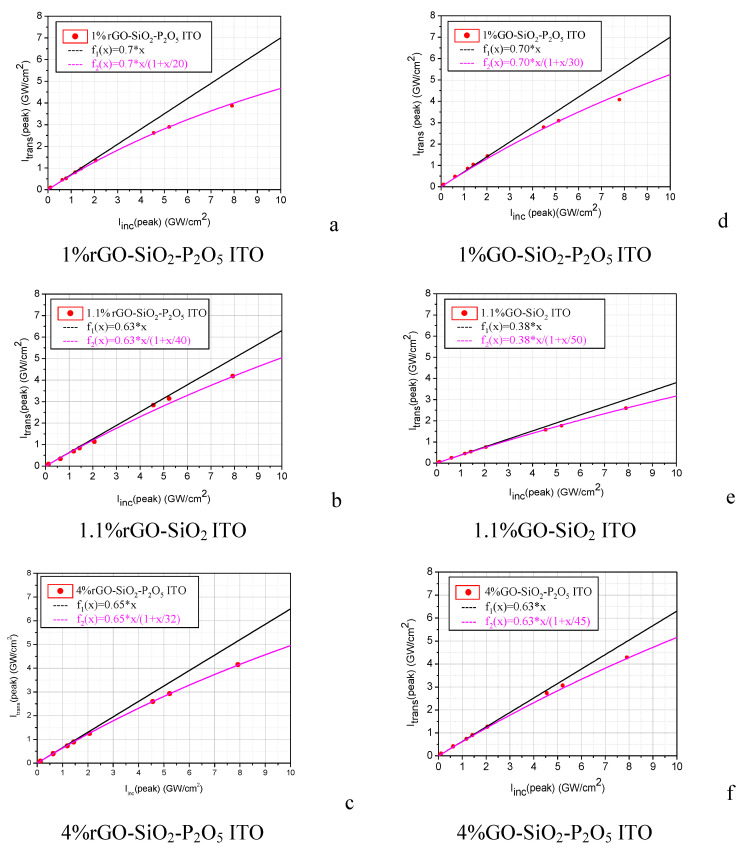
The experimental transmittance results for the films deposited on ITO-coated glass substrates, for the two categories of the investigated silico-phosphate composite samples: with rGO (**a**–**c**) and with GO (**d**–**f**).

The experimental transmittance results, considering the incident, $I_{inc}(peak)$, and the transmitted, $I_{trans}(peak)$, peak intensities of the laser pulses, are shown in Figure 19a–f, for the films deposited on ITO-coated glass substrates for the two categories of the investigated silico-phosphate composite samples, with rGO (Figure 19a–c) and with GO (Figure 19d–f), respectively.

Both the linear fit (considering the experimental points at low incident intensity; linear transmittance) and the fit with the saturation-type function from Equation (1) (considering the entire set of experimental points for each sample; nonlinear transmittance) are shown in Figure 19a–f.

In Table 4 are summarized the linear transmittances, *T_L_*, and the saturation intensities, *I_sat_*, obtained by the fitting of the experimental points. *T_L_* is given by the slope of the linear fit of the experimental points corresponding to low incident peak intensities, and *I_sat_* is obtained from the nonlinear fit of all experimental points with the saturation-type function from Equation (1), respectively.

The comparative analysis of the OL results shown in Figure 19 and in Table 4 reveals that the samples with rGO (Figure 19a–c) limit better (lower *I_sat_*) than the corresponding (the same concentration) samples with GO (Figure 19d–f), for all considered concentrations (1, 1.1 and 4%) of rGO/GO.

From the sets of figures Figure 19a,c and Figure 19d,f, respectively, it is possible to see that the samples with P_2_O_5_ with a lower concentration of rGO/GO (1%) limit better than the samples with higher concentration of rGO/GO (4%). Additionally, the linear transmittance of the samples with a lower content of rGO/GO is higher than that of the samples with a higher content of rGO/GO. Regarding the linear transmittance of the samples with the same concentration of rGO/GO, it is practically similar, excepting the samples without P_2_O_5_ (Figure 19b,e). In this last-mentioned case, the lower linear transmittance of the sample with GO compared to that of the one with rGO could be attributed to the larger content of H_2_O that can be hydrogen bonded to the OH moieties from the surface of GO.

The comparative analysis of all the results shown in Figure 19a–f also reveals the favourable effect on the OL capability of the phosphoric acid used in the preparation of the sol-gel matrix. H_3_PO_4_ contributed to the properties of the films in several ways: (i) the graphenization of GO as a result of the water elimination reaction in the presence of H_3_PO_4_ [14]; (ii) the modification of the interlinkage of the rGO sheets (collective strength of interlayers) due to the modification of the network of hydrogen bonds mediated by oxygen-containing functional groups and water molecules and, accordingly, the change in the materials’ properties [48]; (iii) a more homogeneous distribution of the rGO sheets in the precursor mixtures and in the obtained films.

The OL trend in the silico-phosphate glassy films with rGO on the ITO-coated glass substrate is similar to that reported in isolated fullerene-rich thin films at the wavelength of 532 nm [57]. In our case, the onset of OL (defined as the point on the transmittance curve at which it starts to diverge from the linear transmittance [19]) is much lower (<0.5 mJ/cm^2^) than the values (60–140 mJ/cm^2^, for different samples) reported in [57].

The OL trend in our samples is also comparable to those reported in aqueous GO suspensions at 1064 nm, for 35 ps and 4 ns laser pulses [19]. The onset of OL is again much lower in our case than the ones (~0.50 J/cm^2^) reported for the two pulse durations.

The minimum value of the normalized transmittance, obtained by dividing the transmittance ($I_{trans}(peak)$/$I_{inc}(peak)$) corresponding to the maximum incident intensity by the lowest, linear transmittance is in the case of the sample 1%rGO-SiO_2_-P_2_O_5_ ITO equal to ~0.67, which is similar to that obtained in non-covalent functionalized rGO and rGO functionalized with various concentrations of Ag nanoparticles (~0.65), at 800 nm wavelength with 100 fs laser pulses [25]. The minimum normalized transmittances reported in [25] were obtained at incident intensities of ~2 × 10^17^ W/m^2^, a value that is orders of magnitude higher than our maximum incident peak intensity, 8 × 10^13^ W/m^2^.

The normalized transmittance reported by Ren et al. [24] in electrochemical GO samples, at 800 nm wavelength, with 85 fs laser pulses, reached a minimum value of 0.88 at the incident fluence of 200 mJ/cm^2^ and of 0.67 at the incident fluence of 400 mJ/cm^2^. In our sample 1% rGO-SiO_2_-P_2_O_5_ ITO, the minimum normalized transmittance ~0.67 was obtained at a much lower incident fluence of ~1.3 mJ/cm^2^.

**Figure 20:**
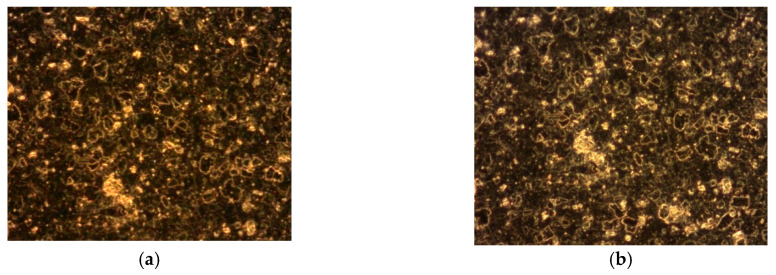
The images of the 1%rGO-SiO_2_-P_2_O_5_ sample, deposited on glass, obtained by differential interference contrast (DIC) microscopy (**a**) before exposure to laser radiation and (**b**) after exposure to laser radiation with *I_inc_(peak)* = 7.7 GW/cm^2^.

In the range of the incident peak intensities from our I-scan experiments, no optical damage was observed in the investigated samples, of which surface was visualized by microscopy before and after exposure to laser radiation (Figure 20).

The surface of the investigated samples was analysed with an optical microscope (Axiotech Vario microscope, Carl Zeiss, Jena, Germany) using DIC microscopy.

## 4. Conclusions

We have demonstrated the preparation of graphene oxide-based silico-phosphate composite films for the optical limiting of ultrashort (fs) laser pulses in the NIR spectral domain, by a low cost and environmentally friendly sol-gel method.

The FTIR spectra revealed the H_3_PO_4_ action to lower the concentration of oxygen-containing functional groups, mainly –OH, from the rGO/GO structure, presumably via the formation and elimination of H_2_O, followed by a vertical re-agglomeration of the rGO sheets as a result of the change from hydrophilic to hydrophobic character.

A more homogenous distribution of pores for the 1%rGO-SiO_2_-P_2_O_5_ films in the AFM distribution histograms was noticed. For the films deposited on ITO, the roughness was almost similar for the samples with GO and rGO regardless of the presence of phosphorus pentoxide. H_3_PO_4_ contributes to the homogeneity distribution of rGO as noticed from SEM/EDX investigations.

Samples containing rGO/GO deposited on the glass substrate exhibited a transmission as high as 80% for λ higher than 800 nm, and the transmission decreased with increasing rGO/GO content. The phosphorus pentoxide content in the films did not induce a higher absorption. The transmittance of all the samples followed the trend line of the ITO/glass substrate with T = 80% up to 1100 nm. For 1100–1800 nm, the increase in rGO content and the presence of phosphorus pentoxide induced a decrease in the transmittance. For the films on ITO/glass, containing 1%rGO, the transmittance did not decrease under 60%.

The Raman spectra revealed an increase in the D band intensity in samples containing P_2_O_5_ as compared with that for the films without P_2_O_5_, meaning a decreased concentration of the groups responsible for the intensity of the D band under the action of H_3_PO_4_. These are consistent with the FTIR and AFM investigations and support the fact that H_3_PO_4_ contributes to a further reduction of rGO, with the rGO sheets uniformly distributed on the substrate.

The optical limiting capability of the graphene-based silico-phosphate composite films was revealed by intensity-scan type experiments. A comparative analysis of the presence and of the concentration of rGO or GO in the structure of the composite glassy films, of the influence of the silico-phosphate matrix, and of the substrate on which the films are deposited on the optical limiting performance of the novel graphene composite films was performed. A comparison of the OL in our samples with several OL results obtained in the literature with ns, ps and fs laser pulses at visible and near infrared wavelengths was performed.

## Figures and Tables

**Table 1:** Composition and denomination of samples.

Sample Denomination	Dopant Material	Matrix Composition	
		SiO_2_/P_2_O_5_ (wt. %)	(rGO or GO)/Ʃ (SiO_2_ + P_2_O_5_) (g/100 g)
1%rGO-SiO_2_-P_2_O_5_	rGO	60/40	1
1.1%rGO-SiO_2_	rGO	100/0	1.1
4%rGO-SiO_2_-P_2_O_5_	rGO	60/40	4
1%GO-SiO_2_-P_2_O_5_	GO	60/40	1
1.1%GO-SiO_2_	GO	100/0	1.1
4%GO-SiO_2_-P_2_O_5_	GO	60/40	4

**Table 2:** Summary of absorption bands in the FTIR spectra of the investigated samples.

Wave Number (cm^−1^)	1.1%rGO-SiO_2_	4%rGO-SiO_2_-P_2_O_5_	4%GO-SiO_2_-P_2_O_5_	SiO_2_-P_2_O_5_	Assignment
3400	Broad band	Broad band	Broad band	Broad band pronounced	υ(O–H) from H–OH adsorbed, C–OH, CO–OH, Si–OH, P–OH
≈1650	-	Less intense	-	Broad band	(O–H) vibrations of water molecules attached to P–O and GO/rGO bonds, υ (C=C)
~910 Shoulders at: ~1060 ~1100 ~1200	Broad band with shoulders	Broad band with shoulders	Broad band with shoulders	Broad band with shoulders	Si–OH stretching with shoulders: υ_as_ (TO)Si–O–P υ_s_ (TO)Si–O–Si υ_s_ (LO) Si–O–Si + υ_s_ O–P–O
768	Pronounced band	Pronounced band	Pronounced band	Pronounced band	υ_s_ (Si–O–Si)

**Table 3:** The root-mean-squared roughness (Rq) and peak-to-valley (Rpv) from atomic force microscopy (AFM) investigations.

Sample	Rq–Rpv (nm) for 20 µm Square Surface	
	On glass	On ITO-coated glass
1%rGO-SiO_2_-P_2_O_5_	15–157.5	2.4–100.3
1.1%rGO-SiO_2_	45–729	2.2–70.5
4%GO-SiO_2_-P_2_O_5_	31–461.2	2.25–70.5

**Table 4:** The linear transmittance and the saturation intensity derived from Figure 19.

Sample Denomination	Linear Transmittance (%)	*I_sat_* (GW/cm^2^)
1%rGO-SiO_2_-P_2_O_5_ ITO (Figure 19a)	70	20
1.1%rGO-SiO_2_ ITO (Figure 19b)	63	40
4%rGO-SiO_2_-P_2_O_5_ ITO (Figure 19c)	65	32
1%GO-SiO_2_-P_2_O_5_ ITO (Figure 19d)	70	30
1.1%GO-SiO_2_ ITO (Figure 19e)	38	50
4%GO-SiO_2_-P_2_O_5_ ITO (Figure 19f)	63	45
